# Qualitative exploration of barriers and facilitators of dental service utilization of pregnant women: A triangulation approach

**DOI:** 10.1186/s12884-018-1773-6

**Published:** 2018-05-10

**Authors:** Hoda Bahramian, Simin Z. Mohebbi, Mohammad Reza Khami, Rocio Beatriz Quinonez

**Affiliations:** 1 Research Center for Caries Prevention, Dentistry Research Institute, Tehran University of Medical Sciences, Tehran, Iran; 2 Department of Community Oral Health, School of Dentistry, Tehran University of Medical Sciences, Tehran, Iran; 3 Departments of Pediatric Dentistry and Pediatrics, Schools of Dentistry and Medicine, University of North Carolina at Chapel Hill, Chapel Hill, N C USA

**Keywords:** Qualitative research, Oral health, Dental health perceptions, Pregnant women

## Abstract

**Background:**

Pregnant women are vulnerable to a wide range of oral health conditions that could be harmful to their own health and future child. Despite the usefulness of regular dental service utilization in prevention and early detection of oral diseases, it is notably low among pregnant women. In this qualitative study, we aimed to explore barriers and facilitators influencing pregnant women’s dental service utilization.

**Methods:**

Using a triangulation approach, we included pregnant women (*n* = 22) from two public health centers, midwives (*n* = 8) and dentists (*n* = 12) from 12 other public centers in Tehran (Iran). Data was gathered through face-to-face semi-structured interviewing and focus group discussion methods. The analysis of qualitative data was performed using conventional content analysis with MAXQDA10 software.

**Results:**

Reported barriers of dental service utilization among pregnant women were categorized under emerging themes: Lack of knowledge and misbelief, cost of dental care, physiological changes, fear and other psychological conditions, time constraint, dentists’ unwillingness to accept pregnant women treatment, cultural taboos and lack of interprofessional collaboration. Solutions proposed by dentists, midwives and pregnant women to improve dental care utilization during pregnancy were categorized under three themes: Provision of knowledge, financial support and establishing supportive policies.

**Conclusions:**

Understanding perceived barriers of dental service utilization during pregnancy can serve as baseline information for planning and formulating appropriate oral health education, financial support, and legislations tailored for lower income pregnant women, midwives and dentists in countries with developing oral health care system.

## Background

Pregnant women are vulnerable to a wide range of oral health problems including dental caries, periodontal diseases and erosion. These changes can be associated with physiological increase in estrogenic hormones levels, poor oral hygiene practices, microbial changes in oral flora, daily diet alterations, frequent snacking, and vomiting [1–4]. Poor oral health during pregnancy has been associated with increased risk of pre-term birth, low birth weight and clinical manifestations of pre-eclampsia [4–6]. Furthermore, poor oral health can affect nutritional status and quality of life of the pregnant woman and her fetus [7], and can contribute to the development of future early childhood caries in their offspring [8].

Regular dental service utilization can assist in early detection of oral problems and improve oral health status of the population [9]. Unfortunately, the use of dental services among pregnant women is notably low even in developed countries, such as the United States (23–35%) [10], United Kingdom (33–64%) [11], Australia (about 30%) [12], and Greece (27%) [13]. An Iranian study by Hajikazemi et al. showed that 70% of pregnant women had negative attitudes about dental care during pregnancy [7]; and in a study by Shamsi et al. [14], only 37% of Iranian women had dental visit during this period. This low dental attendance is mainly limited to acute dental needs [13]. However, in some countries such as Denmark, dental service utilization of pregnant women has been reported to approximate 90% [15].

Most common reported barriers deterring individuals from receiving dental care include high dental treatment costs and probable lack of insurance, lack of perceived dental treatment need, and time constraints of individuals to arrange time for dental attendance [5, 9]. For pregnant women, additional barriers have been suggested including concerns about fetal safety during dental procedures, beliefs about the unavoidable effects of pregnancy on dental health and lack of awareness about the importance of good oral health during pregnancy. Besides, dentists and prenatal health care providers may be unwilling to perform dental care during pregnancy, hindering oral health care in pregnant women [16, 17]. Hamissi et al., [18] reported, 61% of pregnant women mentioning not being advised by their health care providers to visit a dentist during pregnancy.

In some countries, oral health for pregnant women has been integrated into public health services and primary health care [19–21]. Despite such policies developed to increase dental attendance during pregnancy, women still show low dental service utilization worldwide [10–12, 20, 22, 23] with limited information on the underlying factors that deter women from dental attendance during this period. In this qualitative study, we aimed to better understand barriers influencing pregnant women’s dental service utilization and the context that may facilitate their attendance by gathering pregnant women’s perspectives, and midwives’ and dentists’ assumed to be involved in providing oral health care for the pregnant women in public health centers.

## Methods

### Study design

We used a qualitative method to gather information on underlying barriers and facilitators of dental service utilization during pregnancy. To provide a comprehensive perspective, we took advantage of a triangulation of data sources and approaches, [24] employing face-to-face in depth semi-structured interviewing and focus group discussion methods for pregnant women, midwives and dentists, separately.

### Study site and participants

Tehran is the capital of Iran and the largest city in Western Asia with a population approximating 8.4 million of diverse cultural and social backgrounds. The public health sector comprises 115 centers under supervision of three main medical universities namely, Shahid Beheshty, Iran and Tehran University of Medical Sciences. These centers consist of units for general, oral, occupational, environmental, school, and family health where maternal and child health assessments are performed.

The first part of the study targeted pregnant women (*n* = 22) attending family health units of public health centers for routine prenatal visits in two different districts of Tehran city (one in an affluent and other in a non-affluent area) in 2015. Inclusion criteria included healthy pregnant women obtaining care at the public health centers. Nearly half the women were multiparous and at least 12 weeks of gestational age. Firstly, ten in-depth individual semi-structured interviews were conducted conveniently in ten separate days. Afterwards, two organized focus group discussions, pregnant women focus group discussion 1 [pwfgd 1] and 2 [pwfgd 2] took place. Each group involved six women (*n* = 12) at mentioned centers during a 2-week period. Pregnant women were invited for the focus group discussions by telephone 1 week prior to the session and reminded the day before.

To achieve the triangulation approach, two more focus group discussions were conducted (dentists focus group discussion 1 [dfgd 1] and 2 [dfgd 2]) each involving six dentists (*n* = 12), who were working in 12 health centers in different districts of Tehran. A separate focus group discussion was conducted with participation of midwives (*n* = 8), as midwives focus group discussion (mfgd). Dentist and midwives were selected from those attending their monthly meetings in health networks. The interviews and focus group discussions were completed between January and February 2015.

### Interview content and data collection process

Interviews were based on topic guides, including a series of open-ended questions considered to explore and probe the interviewees. All interview guide questions for pregnant women, midwives and dentists were finalized after pretesting in two pilot individual interviews of each group outside the study population and extracting their code for content analysis.

The semi-structured interviews and group discussions included demographics and pregnancy related history. During the main discussion, pregnant women were asked to describe their experience of dental caries and periodontal diseases before and during pregnancy. The history of receiving dental care was asked to obtain their pattern of dental care and whether they had routine dental visits. They were requested to explain the reasons when no visits were reported during pregnancy and what solutions could facilitate receiving dental service (See Table 1).

**Table 1:** Topics and questions of semi-structured guide applied for pregnant women interviews and focus group discussions

Topic	Questions
Demographic characteristics	*How old are you?* *What is your educational level?*
Pregnancy related characteristics	*How far are you in your pregnancy?*
Experience of dental caries and periodontal diseases before and during the pregnancy	*“How would you describe your dental and oral status before and during pregnancy?”*
History of receiving dental care	*“How often do you usually visit the dentist and when was your last dental visit?”*
Beliefs about dental care during pregnancy	*“What is your opinion about visiting the dentist or dental procedures during pregnancy?”*
Reasons if no visits were reported during pregnancy and what solutions could facilitate receiving dental services	*“What causes you to neglect dental checkup during pregnancy and what factors can facilitate it?”*

We modified the questions when interviewing health professionals to obtain their points of view regarding barriers and facilitators of dental utilization or dental service delivery to pregnant women. Midwives and dentists were asked whether or not they had taken any particular courses about prenatal oral health care during study years and their current oral health care provision when delivering care to pregnant women.

All interviews and focus groups were held at an available quiet room commonly used for group education in public health centers. The chairs were arranged in a roundtable, making eye contact of participants possible during focus group discussions. Two chairs were put in front of each other for individual interviews. A dentist who was trained in qualitative research methods as a moderator conducted semi-structured interviews. Other than the moderator, an assistant participated in sessions to organize participant’s time, speaking order, and taking notes in role of facilitator. The assistant was an experienced midwife previously trained in prenatal oral health.

During all sessions, participants were provided a meal during break time. A clinical examination by a dentist and a toothbrush, toothpaste and oral hygiene instruction pamphlet were provided. The midwife then advised participants on pregnancy issues, related oral health problems and answered remaining questions. For the midwives’ and dentists’ sessions, participants received updated oral health information on dental care tips during pregnancy, and a package of toothbrush and toothpaste. Midwives were also examined for their own oral status, while no examinations offered for the dentists group.

Data collection was stopped when data saturation was reached, meaning that no new themes or ideas were being generated during the discussions. This was determined by ongoing analysis of interviews and obtaining no new data while confirming preliminary codes. Interviews were conducted in Persian and transcripts translated into English by a credentialed translator of Tehran University of Medical Sciences.

### Data analysis

The analysis of qualitative data was performed using conventional content analysis for primary code stratification from the text data. All interviews were audiotaped and transcribed verbatim with participants’ consent. Data analysis began with the first interviews. Interviews were reviewed and coded using MAXQDA10 software [25] for pregnant women, dentists and midwives, separately. We applied an inductive approach, with two researchers independently extracting codes from the interviews and focus group discussions in the two study topics (barriers and facilitators). Codes with most propinquity were categorized as subthemes, with main themes consisting of several subthemes [26].

### Methodological considerations

Credibility and conformability was enhanced through member checking (in this case, the transcripts and codes extracted from the interviews were returned to several interviewees to verify their authenticity). Data was de-identified and validation of emerging codes and categories debriefed by a second researcher and their work compared to ensure inter-coder reliability. Less than 5% variation in coding was resolved through discussion. All transcripts, codes, subthemes and theme categories were rechecked, with full agreement achieved among the study team and reviewer.

## Results

Data was collected on 22 pregnant women, 12 dentists and 8 midwives regarding their perspective views on barriers of dental service utilization for pregnant women. Fifteen women refused to participate in the study and all dentist and midwives consented to participate. Pregnant women’s age ranged from 18 to 45 years, with a mean of 30.4 (± 7.6) years. Gestational age ranged from 16 to 36 weeks, with a mean of 25.3 (± 6.4) weeks. Thirty-two percent had more than high school education and 14% were illiterate. The mean dentists and midwives age were 36.6 (±9.6) and 37.9 (±6.2), respectively. About 85% of midwives and 60% of dentists expressed they had not taken any continuing education courses regarding prenatal oral health.

The time duration of interviews and focus group discussions ranged from 35 min to 1 h and a half with the following themes emerging:

Lack of knowledge and misbelief about dental care, cost of dental care, physiological changes, fear and other psychological conditions, time constraint, dentists’ unwillingness to accept pregnant women treatment, cultural taboos and lack of interprofessional collaboration. They are listed below from the most to the least frequent mentioned barrier per participant type. Lack of knowledge and misbelief followed by cost of dental treatment were the most common barrier reported by all three groups.

### Lack of knowledge and misbelief about dental care

#### Pregnant women

1. Although higher educated and multiparous pregnant women showed better knowledge and attitude regarding dental visits during pregnancy, many pregnant women were unaware of the significance of preventive dental visits before and during pregnancy.

- *“I had checkup examinations for my whole body health and took blood test and visited obstetrics physician but I did not visit a dentist. Nobody told me it is a must, even my physician. I was not informed about that.” (interview 5)*
- *“I do not perceive a need to visit a dentist as all things are good about my teeth and oral condition, and I inherited strong teeth from my father.” (interview 7)*
- *“As I am consuming iron and calcium supplements during pregnancy, I think my teeth are less likely to be decayed because the supplements will compensate calcium deficiency during pregnancy so I do not have to visit the dentist in this period.” (interview 4)*

2. Some women felt defenseless against dental caries in pregnancy and assumed it an expected part of life.

- *“I believe that in every pregnancy at least one tooth is destructed, because calcium from the mother’s teeth is consumed for formation of fetus skeleton. I myself experienced it in my first pregnancy, all my teeth were sound before pregnancy but after delivering my baby, I noticed a cavity in one of my teeth.” (interview 5)*

3. The false belief that dental treatment during pregnancy is unsafe was among the most important reasons limiting access to dental care during pregnancy.

- *“I usually try to tolerate dental pain and not go to the dental clinic, as dentist prescribe medicine, so I do not go and do not take medicine that might be harmful to my baby.” (pwfgd 2)*
- *“I heard that radiography and anesthesia injection can cause miscarriage, so I am afraid of visiting the dentist during pregnancy.” (interview 5)*

### Dentists and midwives

Dentists and midwives also decisively mentioned the lack of knowledge among pregnant women about the importance of preventive dental visits before and during pregnancy. Safety of dental treatment during this period was also mentioned, which may decrease women’s dental utilization. Furthermore, providers also reported insufficient knowledge and competency of dental service delivery for pregnant women.

- *“I remember during school years we had just briefly learned about oral health care for pregnant women in the community dentistry course and as limited topics in emergencies in endodontic and oral surgery field. But in my opinion it was an incomplete education as it didn’t give us enough self-confidence and knowledge because there was no regular specific course in the curriculum regarding oral care of pregnant women” (dfgd 1)*
- “*I myself am not sure about safety of dental procedure for pregnant women, so I prefer to avoid it. Most patients request guarantee that no risk will threaten their fetus, but we cannot give such a guarantee to them.” (dfgd 1)*
- “*I do not know exactly the legal rules in Iran for dental care for pregnant women and probable problems during treatment, but I know that the written consent form of obstetric physician is necessary and also a consent form of the patient and her husband.” (dfgd 1)*

Midwives also pointed to lack of formal and organized training on oral health issues, emphasizing that oral health education was not taught in their midwifery training courses. They lacked confidence in advising pregnant women on this topic and subsequently motivating patients to visit the dentists [27].

- *“It is important for midwifes to know primary signs of dental caries and diagnose them properly. But we were not trained sufficiently in this field. For instance, I myself still cannot distinguish between the black lines on my teeth as caries, tea or coffee stains, let alone diagnosis of caries in my patient’s mouth.” (mfgd)*

### Cost of dental care

The majority of pregnant women (95%) indicated that their neglect for regular dental visit was due to high dental service costs and incomplete dental insurance coverage.

- “*I myself was referred to a dentist before pregnancy for a checkup, but the expenses were too high for me. I just had my emergency dental treatments done in order to prevent dental pain during pregnancy.*” *(interview 8)*
- *“I do not receive services that my dental insurance won’t pay for because the cost of dental treatments is very high for me and my annual ceiling insurance is limited.” (interview 5)*

Midwives and dentists also pointed to the cost of dental services as the most critical barrier in respect to not seeking dental care.

- *“The first important barrier is cost and the last is also cost and cost and cost. I myself as a midwife who is involved in health issues, 2 years ago when I was pregnant, I had not visited the dentist because of the fear of high expenses for probable treatment needs.” (mfgd)*
- *“Many pregnant women, who come for dental checkup, cannot afford for dental fees.” (dfgd 1)*

### Physiological changes

#### Pregnant women

Physiological alterations in pregnancy, including nausea, limitation in movements, and sitting in the dental chair could be one of reasons that pregnant women less desire to attend the dentist.

- *“I cannot tolerate any external thing in my mouth, even my toothbrush makes me have gag reflex so the dentist won’t be able to work on my teeth.” (pwfgd 2)*
- “*I have had a backache since I gained weight during pregnancy so I could not sit a long time and lay in a back position like usual in the dental chair*” *(pwfgd 1)*

#### Dentists and midwives

- “*Dental treatment should be done as fast as possible for pregnant women because sleeping on their back is lengthy in the dental chair and can put pressure on the Inferior vena cava and should be avoided. Sometimes maybe this is difficult to do with some dental procedures in limited amount of time.” (mfgd)*
- *“Severe nausea during pregnancy in some cases limits us using dental instruments inside the mouth and performing dental procedures” (dfgd 2)*

Lack of modern and new equipment hampers the ability to provide expediently dental care for pregnant women.

- *“We have just an old and uncomfortable dental chair and some old dental hand pieces that do not work properly and slow down the dental procedures, in contrast with misconceptions to treat pregnant women as fast as possible.” (dfgd 1)*

Considering physiological changes in pregnant women, emergencies are more likely to occur during dental treatments, which health care providers should be prepared for.

- *“There are no hospital facilities for rescue in our health center in case of emergencies” (dfgd 2)*

### Fear and other psychological conditions

1. Some pregnant women mentioned not visiting the dentist during pregnancy because of overall fear and anxiety about going to dentist, regardless of this pregnancy period.

- *“I have been afraid of dentists since my childhood; going to the dentist always makes me stressed and accelerates my heartbeat” (interview 1)*

2. Feeling boredom, laziness and impatience during pregnancy results in decreased chance of dental visits during this period.

- *“I have become lazy and impatient by starting this period and I do not like to leave home for hours to attend the dentist and wait for my dental turn.” (pwfgd 2)*

3. Unexpected and consecutive pregnancies make women depressed and careless about their health.

- “*Pregnant women who did not want to be pregnant usually become depressed and unresponsive to their health and subsequently they seldom seek the dentist.” (mfgd)*

### Time constraint

#### Pregnant women

1. Some pregnant women were too busy to arrange time off from work to have routine dental visit.

- *“I can afford the expenses of dental services somehow and my insurance company to some extent has annual good support. But I cannot arrange my time to attend dental appointments because I am too busy and I cannot recess from my workplace.”* *(interview 6)*

2. In some cases, having a large family size and looking after previous children prevented them from making time to visit the dentist themselves.

- *“I have two naughty boys and no one can look after them while I want to go everywhere.” (pwfgd 2)*

#### Midwives

Limitation of time in midwives’ work schedule results in neglecting screening and referral for dental care during pregnancy. The beliefs that oral health is not their main duty and current patient overload, does not make oral examination task a priority of care.

- *“Our health center is very crowded, we have to visit and consult over 30 pregnancies daily so how can I find more time to pay attention to my patients’ teeth to refer them or extend my consultation through oral health?” (mfgd)*

### Dentists’ unwillingness to treat pregnant women

#### Pregnant women

It was also indicated that dentists seldom provide dental care services for pregnant women.

- *“During my previous pregnancy, 1 day I experienced severe dental pain and I went to the nearest dental clinic to my home. They did not do anything for me and told me that we have no equipment for treating pregnant women and referred me to the Sajad hospital that provides dental services for pregnant women.” (interview 2)*
- *“I think most dentists are afraid of offering care for pregnant women so referral for written permission from a gynecologist is needed.” (pwfgd 1)*

#### Dentists

Perceived lack of support from authorities for dentists caring for pregnant women in case of occurrence of unwanted problems while caring for pregnant women, result in unwillingness to treat these groups by most dentists. As one of them said:

- “*Hmmm, I know that caring for pregnant women is not a guilt or crime but I am afraid of treating them as there are thousands of reasons for miscarriage like, electromagnetic waves and parasites, air pollution, water pollution, … but all people blame dentist if something bad would happen.” (dfgd 1)*

#### Midwives

Some midwives emphasized that they refer pregnant women for dental care during pregnancy, but dentists are unwilling to provide this care, particularly for low-income pregnant women.

- *“We refer pregnant women to the dentists routinely but the dentists don’t accept them for treatment and postpone treatment until after labor.” (mfgd)*

### Cultural taboos

Some cultural taboos and religious bias have prevented individuals from getting appropriate care from health care providers.

- *“Our family believes in traditional and herbal medicine and thanks God until now we have not needed to visit a dentist.” (pwfgd 1)*
- *“Extracting teeth roots as missing an organ of the body must be avoided. But dentists do not leave them in the mouth although they do not bother me.” (pwfgd 2)*
- *“My husband does not permit me to visit a male dentist and most available and qualified dentists who care for pregnant women are men.” (pwfgd 2)*

### Lack of interprofessional collaboration and supportive policies

#### Dentists

1. Dentists indicated that other health care providers in health centers usually do not cooperate with them in case of emergencies:

- *“In our medical center, physicians do not take responsibility caring for pregnant women. If we encounter any emergencies during a dental procedure, it is our responsibility, so we prefer not to accept pregnant women for dental treatment from the beginning.” (dfgd 1)*

2. A number of dentists indicated they could treat pregnant women confidently but midwives in health centers did not collaborate by referring and educating pregnant women.

- “*I myself have no fear of delivering dental care to pregna*nt *women and I have done several treatments like pulp therapy and … for them. They were satisfied with the treatments and in spite of midwives’ accusation saying they refer and dentists refuse, I should say that some of them do not bother themselves even to write patient name on the referral paper, let alone the oral health education. With several years of education we have both knowledge and experience to care for pregnant women.” (dfgd 2)*
- *“Misdirection of even obstetrics about oral health. For instance, they advise pregnant women not to visit dentists because dental treatment is not safe, for example one of them had prescribed calcium supplements for pregnant women in order to help the patient to prevent dental caries in pregnancy.” (dfgd 2)*

#### Midwives

Lack of supervision on referral to the dentist

- *“There is no strict law for referring pregnant women to dentist for prenatal care. Indeed, there is law and we as midwives refer, but if they did not visit the dentist nothing will happen and they won’t be punished and they receive their subsequent care” (mfgd)*

### Facilitators

The facilitators (solutions) for improving dental care utilization during pregnancy, as proposed by dentists, midwives and pregnant women are listed in Table 2. Most solutions could be classified under three categories of “provision of knowledge”, “financial support” and “establishing supportive policies”. Suggestions included providing educational and financial opportunities, and supportive legislation and environment for pregnant women to seek and access required dental care. These recommendations could be beneficial for improving dental care utilization during pregnancy.

**Table 2:** Solutions for improving dental care utilization in pregnancy proposed by midwives, dentists, and pregnant women

Solution type	Proposed solutions by participants and related quotes		
	Midwives	Dentists	Pregnant women
Provision of knowledge	*“In my opinion the most important barrier for seeking dental visit during pregnancy is lack of knowledge. If someone were informed, he/she would follow. It depends on kind of informing in society but advertisements are effective. If useful advertisements about oral health issue in media were even one thousandth of advertisements of junk foods, it would be effective.”*	*“There is a need to include a special course for dental care during pregnancy in dental and midwifery curriculums in universities.”*	*“Pregnant women should be trained on oral self-care; and the importance of dental visit before, during and after pregnancy must be emphasized to them.”*
	*“Oral hygiene and basic dental courses should be included in our curriculum and we must undergo practical training in areas such as caries detection and diagnosis of gum disease in order to have enough self-confidence to guide patients.”*	*“An agreed clear guideline must be developed for dental treatment during pregnancy.”*	*“Midwives should encourage us to seek dental visit.”*
		*“Providing educational programs during pregnancy regarding dental care is advised.”*	
		*“Education in media regarding dental visit in pregnancy should be developed.”*	
Financial support	*“It must be considered a separate fee for service for dentists to treat pregnant women in order to motivate them for treatment.”*	*“Special discounts or insurance packages should be offered to pregnant women to decrease the financial problems for seeking dentists.”*	*“If it was possible for us to take dental loan or pay dental fees in installments instead of cash, it was great.”*
	*“Increase the coverage and annual allocation of insurance companies for dental services especially for pregnant women.”*		*“Dental insurances should be improved and cover more dental services for pregnant women.”*
	*“There should be a great discount of dental services or even free dental services for pregnant women in governmental centers.”*		*“Dental cost for pregnant women should be decreased.”*
Establishing supportive policies	*“If pregnant women do not visit the dentist despite referral by midwives, they should be deprived from further free prenatal services.”*	*“Equipped dental clinics should be developed specially for treating pregnant women all over the city.”*	*“Dental centers which provide dental care for pregnant women should be increased.”*
	*“It is better to make dental visit obligatory before and during pregnancy like obligation of consumption of supplements before pregnancy.”*		
	*“It should be mandatory to perform scaling for all women who need before and during pregnancy.”*	*“Midwives and nurses and physicians should cooperate which each other more in health centers about pregnant women oral health.”*	
	*“The pregnant women are not suitable choice for oral health education and cooperation for related interventions since they had physiological problems like nausea and …, It is better to call on them after giving birth?”*		*“To recess from work for dental checkups must become easy for employed pregnant women.”*
	*“The dental centers which specially deliver services to pregnant women should be developed and increased.”*	*“The government regulations should support dentists for caring pregnant women.”*	
	*“Psychological counseling during and after pregnancy could help women to pay more attention to their health generally and seeking necessary cares.”*		

## Discussion

This qualitative study focused on barriers and facilitators of dental service utilization and delivery to pregnant women attending public health centers of Tehran city. Pregnancy is a critical time to educate and counsel individuals on all aspect of health, including oral health as this can influence the well-being of a woman herself and the future child. To improve use of dental services, it is now recommended that all pregnant women receive comprehensive oral health education, evaluation and referral to dentists in case of treatment needs, by prenatal care practitioners [28, 29]. Nevertheless, frequency of dental visits among pregnant women have been reported to be less than optimal in several parts of the world. Providing comprehensive health strategies on oral health promotion have been given a higher priority, showing promising results for dental attendance of pregnant women in some countries such as Denmark [15, 30].

Understanding factors that lead pregnant women to underutilize dental care in this period is valuable in formulating future oral health promotion programs for this susceptible population. This work also revealed and confirmed several common barriers and facilitators of pregnant women’s dental attendance from a triangulation approach of midwives, dentists and pregnant women. The perceived barriers for dental service utilization during pregnancy obtained from our study could highlight some new aspects related to the cultural challenges, including taboos and religious subjects. Moreover, we could address integration of oral health care into public health systems by discussing interprofessional collaboration as recommended by WHO, often at the root of the problem in coordinated care during pregnancy [31].

### Lack of knowledge and misbelief

Our study highlighted areas of deficiency in oral health knowledge and beliefs of pregnant women, dentists and midwives during the prenatal period. The most important barriers were lack of knowledge among pregnant women about the importance of dental visits during pregnancy, misbelief about safety of dental care in this period, and lack of perceived need to visit the dentist during pregnancy. These findings are in line with other studies [32, 33], suggesting the importance to integrate oral health topics into prenatal classes, provide culturally and linguistically appropriate care at a literacy level appropriate for understanding the delivered information [34]. Dentists and midwives also reported insufficient knowledge about prenatal oral health care and requested receiving specific training on this issue, also consistent with previous studies [16, 27]. Similar to George et al. [16], many prenatal care providers were uncertain of the safety of dental procedures during pregnancy and were hesitant to treat pregnant women in spite of current guidelines indicating the safety of dental procedures in all trimesters [4, 16, 34–36]. This underscores the importance of incorporating educational opportunities for oral care during pregnancy into midwifery and dental curricula. Furthermore, emphasizing current guidelines on oral health counseling, screening and referral to a dental home during pregnancy should be a focus of prenatal visits. The importance of parity is noteworthy, with multiparous women reporting being more knowledgeable and having more positive attitude regarding oral health care and importance of regular dental visit. Although having more children may pose a challenge for women to arrange time and child care for regular dental visits, multiparous mothers may have already been exposed to previous oral health instructions [37].

### Cost of dental care

Cost was a common reported barrier to receiving dental care, suggesting consideration be given to the financial implications of accessing care, transportation, childcare and opportunity costs. Cost may also be a barrier even in countries with full dental insurance coverage for pregnant women. To promote oral health of pregnant women and their offspring, discounts should be considered for dental treatments in health centers that include primary and secondary prevention such as scaling and less complex restorative treatments. Midwives, dentists and pregnant women participating in our study suggested that payment for dental services in installments, establishment of dental care loans, or better insurance coverage should be provided. They also suggested that facilities serving low-income pregnant women provide access to dental clinics and services at the lowest possible cost. Awareness of subsidized fees, however, has not been always associated with higher dental attendance of pregnant women [38]. Alternate strategies to address these barriers should include establishing partnerships with community-based programs that serve low-income pregnant women and facilitate insurance application processes and other sources of coverage, provide social services, and fulfill their other needs (e.g., transportation) [34]. Policy considerations to including broader insurance coverage may take advantage of the vast private sector potential that provides approximately 90% of oral health care in Iran.

### Time constraint and social capital

Time constraint for delivering dental care by health care providers was stated as another perceived barrier that accords with other studies [5, 17, 27]. Although health centers are usually busy, health care providers should prioritize main tasks, assigning a short time to examine the clients’ mouths as a component to a history and physical assessment, and refer to the dentists as needed. Counseling on general health messages that overlap with oral health (e.g. nutrition, smoking and alcohol cessation, prenatal vitamins) should not be missed and can minimize time issues as these topics are already being discussed as part of prenatal care.

Time constraints were also mentioned by pregnant women as one of the barriers for receiving dental care. Le et al. in [17] emphasized the role of social and family support for having positive health behaviors during pregnancy, including regular dental visit. One of the participants mentioned the problem of finding care for her elder child when seeking dental care during pregnancy. Preparing children’s playroom in health centers and offering no cost or low cost child care services during women’s visits (both prenatal and dental) may be a useful solution to increase health care utilization for pregnant women with lower social capital.

### Lack of interprofessional collaboration

The concept of integrated health service delivery has been emphasized by the WHO in response to resource constraints; in particular human resource deficiency in low-income countries. Accordingly, health professionals should collaborate and refer patients to each other to provide comprehensive quality care [31]. The Iran Oral Health Care System targeting mothers and children has been integrated into the public health system since 1997. The integration needs cooperation of general health care providers and dentists. Although progress has been made, shortcomings still exist, making the health system far from ideal [20]. As shown in our study, health providers (midwives and dentists) usually blamed each other for the lack of referrals and cooperation, thus compromising coordinated care during pregnancy.

In concordance with the results of Klotzel et al. (2011) [33] and George et al. [16], lack of interprofessional collaboration in some health centers of Tehran may discourage dentists from treating pregnant women. Both beliefs among health care providers that screening and referral for dental care during pregnancy is not their main duty and current patient overload precludes oral screenings for pregnant women from occurring [16, 33]. It is recommended that legislating laws mandate interprofessional collaboration to refer, support or supplement care between health professionals regarding general health and oral health during pregnancy [39]. Developing formal referral processes that are easily trackable via electronic patient records, will allow for monitoring of timely referrals and delivery of necessary oral health services [34]. Quality improvement initiatives can facilitate this process and promote sustainable oral health interventions during pregnancy [40].

### Physiological and psychological changes

Despite the perception among pregnant participants that physiological changes and alterations in their psychological conditions during pregnancy limits receiving dental care, these conditions are routine and unavoidable. Accordingly, it is advised to increase women’s awareness of the normality of these changes, and to encourage dental treatment before becoming pregnant to avoid these and other limitations in receiving care during pregnancy. Therefore, dental visits before pregnancy are very important to inform probable dental care needs and learning positive oral health behaviors for child bearing age women.

### Cultural taboos

Some participants stated that receiving dental services delivered by male dentists was undesirable given the religious aspects of different gender delivering care. For some women, the hesitation that male dentists provide dental treatments emerged from their spouses showing bias type behaviors. Although Islam does not ban treatment by the opposite sex, providing the patient with a dentist or physician of the same sex when possible is recommended, especially if the patient feels strongly about it [41]. Conversely, some participants believed that male dentists had greater expertise and skill sets than females. Despite female dentist availability in health centers, other health professionals did not refer to them. Providing access to more male and female dentists available in the private sector may help patients selecting on gender preference in the short term. Changing cultural norms and mores is more difficult and may be assumed as a long-term goal.

### Facilitators

Facilitators of pregnant women’s dental attendance was emphasized by the reported need for intensive education on prenatal oral health programs, provision of financial support, and legislations for full establishment of integrated health care systems in Iran. The desired increase in oral health education is confirmed by recent work revealing Iranian primary care providers’ willingness to learn more and deliver oral health care to their patients [42, 43]. It is notable that in our study midwives and dentists also provided suggestions on ways to increase their responsibility regarding oral health care for pregnant women. When examining the larger Iranian heath care system, some suggested solutions to address oral health during pregnancy have already been incorporated, but are not widely disseminated nor perceived to be available services by all participants. Further strategies to promote dissemination and implementation of oral health during pregnancy in the private and public sectors are warranted, and can in part be achieved by modifications on financial structures and mandated legislature of practice guidelines.

### Strengths and limitations

Using the triangulation approach, this qualitative study obtained rich information and deeper understanding of selected participants’ beliefs from some public health centers in both affluent and non-affluent districts of Tehran. We benefited from this approach and used multiple data sources (perspectives of pregnant women, midwives and dentists) to better understand dental utilization challenges during pregnancy and shed light on this phenomenon. As with any qualitative research, however, it is impossible to generalize data to entire populations of women, midwives and dentists; with further cross sectional surveys to evaluate all perspectives is warranted. A further limitation includes the lack of interviews with mothers who did not attend public health centers, specifically those of higher socioeconomic status, therefore limiting our generalizability to whole pregnant women. Nevertheless, most health problems and challenges are more common among disadvantaged individuals in the communities. Finally, the limitations that are inherent in bias of excluding pregnant women who refused to participate because of personal disagreement with voice recording, time constraint, or simply being pregnant and not wanting to wait.

## Conclusions

A wide variation of perceived barriers of dental service utilization during pregnancy were derived from this study including lack of knowledge about safe dental care and pregnancy related changes, lack of interprofessional collaboration, economic challenges, and cultural taboos. Understanding the barriers can serve as baseline information for planning and formulating appropriate oral health intervention tailored for lower income pregnant women, midwives and dentists. Targeted educational programs, financial supports and legislations for full establishment of an integrated health care system that promotes high quality coordinated care may provide higher availability of dental services leading to reduced health disparities in countries with developing oral health systems.

## Abbreviations

dfgd: dentists focus group discussion
mfgd: midwives focus group discussion
pwfgd: pregnant women focus group discussion

## References

[1] Abiola A, Olayinka A, Mathilda B, Ogunbiyi O, Modupe S, Olubunmi O (2011). A survey of the oral health knowledge and practices of pregnant women in a Nigerian teaching hospital. Afr J Reprod Health.

[2] Kandan PM, Menaga V, Kumar RR (2011). Oral health in pregnancy (guidelines to gynaecologists, general physicians & oral health care providers). J Pak Med Assoc.

[3] Kurien S, Kattimani VS, Sriram RR, Sriram SK, Rao V K P, Bhupathi A, Bodduru RR, N Patil N: Management of Pregnant Patient in dentistry**.** J Int Oral Health 2013;5(1):88–97. PMC3768073 24155583

[4] Steinberg BJ, Hilton IV, Iida H, Samelson R (2013). Oral health and dental care during pregnancy. Dent Clin N Am.

[5] Buerlein JK, Horowitz AM, Child WL (2011). Perspectives of Maryland women regarding oral health during pregnancy and early childhood. J Public Health Dent.

[6] Daalderop LA, Wieland BV, Tomsin K, Reyes L, Kramer BW, Vanterpool SF, Been JV. Periodontal disease and pregnancy outcomes: overview of systematic reviews. JDR Clin Translat Res. 2017; 10.1177/2380084417731097. 10.1177/2380084417731097 PMC6191679 30370334

[7] Hajikazemi E, Haghdoost Osquei F. Oral and Dental Health in Pregnancy, Oral Health Care - Pediatric, Research, Epidemiology and Clinical Practices, Prof. Mandeep Virdi (Ed.), ISBN: 978-953-51-0133-8, InTech, 2012. https://api.intechopen.com/chapter/pdf-download/29346. Accessed 1 May 2018.

[8] Gussy MG, Waters EG, Walsh O, Kilpatrick NM (2006). Early childhood caries: current evidence for aetiology and prevention. J Paediatr Child Health.

[9] Bahramian H, Mohebbi SZ, Khami MR, Asadi-Lari M (2015). Psychosocial determinants of dental service utilization among adults: results from a population-based survey (Urban HEART-2) in Tehran, Iran. Eur J Dent.

[10] Gaffield ML, Gilbert BJC, Malvitz DM, Romaguera R (2001). Oral health during pregnancy: an analysis of information collected by the pregnancy risk assessment monitoring system. J Am Dent Assoc.

[11] Hullah E, Turok Y, Nauta M, Yoong W (2008). Self-reported oral hygiene habits, dental attendance and attitudes to dentistry during pregnancy in a sample of immigrant women in North London. Arch Gynecol Obstet.

[12] Thomas NJ, Middleton PF, Crowther CA (2008). Oral and dental health care practices in pregnant women in Australia: a postnatal survey. BMC Pregnancy Childbirth.

[13] Dinas K, Achyropoulos V, Hatzipantelis E, Mavromatidis G, Zepiridis L, Theodoridis T, Dovas D, Tantanasis T, Goutzioulis F, Bontis J (2007). Pregnancy and oral health: utilisation of dental services during pregnancy in northern Greece. Acta Obstet Gynecol Scand.

[14] Shamsi M, Hidarnia A, Niknami S (2013). Self-reported oral hygiene habits and self-Care in the Oral Health in sample of Iranian women during pregnancy. World Appl Sci J.

[15] Christensen LB, Jeppe-Jensen D, Petersen PE (2003). Self-reported gingival conditions and self-care in the oral health of Danish women during pregnancy. J Clin Periodontol.

[16] George A, Shamim S, Johnson M, Dahlen H, Ajwani S, Bhole S, Yeo AE (2012). How do dental and prenatal care practitioners perceive dental care during pregnancy? Current evidence and implications. Birth.

[17] Le M, Riedy C, Weinstein P, Milgrom P (2009). Barriers to utilization of dental services during pregnancy: a qualitative analysis. J Dent Child (Chic).

[18] Hamissi J, BakianianVaziri P, Davalloo A (2010). Evaluating oral hygiene knowledge and attitude of pregnant women. Iran J Public Health.

[19] Boggess KA, Edelstein BL (2006). Oral health in women during preconception and pregnancy: implications for birth outcomes and infant oral health. Matern Child Health J.

[20] Pakshir HR (2004). Oral health in Iran. Int Dent J.

[21] Russell SL, Mayberry LJ (2008). Pregnancy and oral health: a review and recommendations to reduce gaps in practice and research. MCN Am J Matern Child Nurs.

[22] Shamsi M, Hidarnia A, Niknami S, Rafiee M, Karimi M (2013). Oral health during pregnancy: a study from women with pregnancy. Dent Res J (Isfahan).

[23] Vamos CA, Thompson EL, Avendano M, Daley EM, Quinonez RB, Boggess K (2015). Oral health promotion interventions during pregnancy: a systematic review. Community Dent Oral Epidemiol.

[24] Adami MF, Kiger A (2005). The use of triangulation for completeness purposes. Nurse Res.

[25] MAXQDA, software for qualitative data analysis, 1989-2018, VERBI Software – Consult – Sozialforschung GmbH, Berlin, Germany. https://www.maxqda.com/faq/how-do-i-cite-maxqda. Accessed 1 May 2018

[26] Hsieh HF, Shannon SE (2005). Three approaches to qualitative content analysis. Qual Health Res.

[27] Heilbrunn-Lang AY, de Silva AM, Lang G, George A, Ridge A, Johnson M, Bhole S, Gilmour C: Midwives’ perspectives of their ability to promote the oral health of pregnant women in Victoria, Australia BMC Pregnancy Childbirth 2015;15(1):1. 10.1186/s12884-015-0536-x PMC4490712 25943399

[28] Hayes DK, Turnure M, Mattheus DJ, Shannon MT (2015). Predictors of dental cleaning over a two-year time period around pregnancy among Asian and native Hawaiian or other Pacific islander race subgroups in Hawai ‘i, 2009–2011. Hawaii J Med Public Health.

[29] Martinez-Beneyto Y, Vera-Delgado MV, Perez L, Maurandi A (2011). Self-reported oral health and hygiene habits, dental decay, and periodontal condition among pregnant European women. Int J Gynaecol Obstet.

[30] Christensen LB, Petersen PE, Steding-Jessen M (2007). Consumption of dental services among adults in Denmark 1994–2003. Eur J Oral Sci.

[31] World Health Organization. Technical brief No. 1 2008. Integrated health services– what and why? 2008. http://www.who.int/healthsystems/technical_brief_final.pdf. Accessed 1 May 2018.‎

[32] George A, Johnson M, Duff M, Ajwani S, Bhole S, Blinkhorn A, Ellis S (2012). Midwives and oral health care during pregnancy: perceptions of pregnant women in South-Western Sydney, Australia. J Clin Nurs.

[33] Kloetzel MK, Huebner CE, Milgrom P (2011). Referrals for dental care during pregnancy. J Midwifery Womens Health.

[34] Oral Health Care During Pregnancy Expert Workgroup. Oral health care during pregnancy: a national consensus statement—summary of an expert workgroup meeting. Washington, DC: National Maternal and Child Oral Health Resource Center; 2012. https://www.in.gov/isdh/files/Oral_Health_Care_During_Pregnancy_Consensus.pdf. Accessed 1 May 2018

[35] Dental X-Rays, Teeth cleanings = safe during pregnancy. 2013. https://www.perioimplantadvisory.com/articles/2013/07/dental-x-rays-teeth-cleanings-safe-during-pregnancy.html. Accessed 1 May 2018.

[36] Huebner CE, Milgrom P, Conrad D, Lee RSY (2009). Providing dental care to pregnant patients. J Am Dent Assoc.

[37] Baker SD, Quiñonez RB, Boggess K, Phillips C. Pregnant Women’s infant oral health knowledge and beliefs: influence of having given birth and of having a child in the home. Matern Child Health J. 2016:1–8. 10.1007/s10995-016-1930-3 26961141

[38] Ahmadian Yazdi A, Sanatkhani M (2004). A descriptive survey of the oral health on a group of the asian pregnant women resident in the UK. J Mashad Dent Sch.

[39] Jackson JT, Quinonez RB, Kerns AK, Chuang A, Eidson RS, Boggess KA, Weintraub JA (2015). Implementing a prenatal oral health program through interprofessional collaboration. J Dent Educ.

[40] Simpson DD (2011). A framework for implementing sustainable oral health promotion interventions. J Public Health Dent.

[41] Guidelines for Health Care Providers Interacting with Muslim Patients and their Families. ‎‎2006. http://www.ispi-usa.org/guidelines.htm. Accessed 1 May 2018.

[42] Rabiei S, Mohebbi SZ, Patja K, Virtanen JI (2012). Physicians’ knowledge of and adherence to improving oral health. BMC Public Health.

[43] Rabiei S, Mohebbi SZ, Yazdani R, Virtanen JI (2014). Primary care nurses’ awareness of and willingness to perform children’s oral health care. BMC Oral Health.

